# Treatment of children with COVID-19: update of the Italian Society of Pediatric Infectious Diseases position paper

**DOI:** 10.1186/s13052-021-01132-2

**Published:** 2021-10-07

**Authors:** Elisabetta Venturini, Carlotta Montagnani, Silvia Garazzino, Daniele Donà, Luca Pierantoni, Andrea Lo Vecchio, Andrzej Krzysztofiak, Giangiacomo Nicolini, Sonia Bianchini, Luisa Galli, Alberto Villani, Guido Castelli Gattinara, the Italian SITIP-SIP SARS-Cov-2 pediatric infection study group

**Affiliations:** 1 Infection Disease Unit, Meyer Children’s University Hospital, Florence, Italy; 2 Pediatric Infectious Disease Unit, Regina Margherita Children’s Hospital, University of Turin, Turin, Italy; 3 Department for Woman and Child Health, Division of Pediatric Infectious Diseases, University of Padua, Padua, Italy; 4 Pediatric Emergency Unit, Policlinico di Sant’Orsola, Bologna, Italy; 5 Department of Translational Medical Science, Section of Pediatrics, University of Naples Federico II, Naples, Italy; 6 Universitarian-Hospital Department, Bambino Gesù Children Hospital - IRCCS, Rome, Italy; 7 UOC Pediatria, San Martino Hospital, Belluno, Italy; 8 Department of Pediatrics, ASST Santi Paolo e Carlo Hospital, Milan, Italy; 9 Department of Health Sciences, University of Florence, Florence, Italy

Dear editor,

The Italian Society of Pediatric Infectious Diseases steering and scientific committee published a position paper on the treatment of children with COVID-19, updated at 16 June 2020 [1].

Since evidences are rapidly evolving and therapeutic indications are changing very quickly, an update of this consensus is considered essential. In fact, it has been observed that during the second and third waves of COVID-19 the rate of affected children is higher, despite it is well known that the majority of children present a pauci-symptomatic course of the disease. This increase could also be related to the diffusion of virus variants across Europe [2]. Therefore, the available literature on pediatric treatment strategies for SARS-CoV-2 infection in children was reviewed up to 10 March 2021 on PubMed, the website of national and international scientific societies and of clinical trials.

Suggested COVID-19 management and treatment in children, according to disease severity, are shown in Table 1. Multi-inflammatory syndrome treatment, including use of anti-cytokine treatments, is not discussed in this paper, as specific Italian guidelines have been recently issued [5].

**Table 1:** COVID-19 management and treatment in children, according to disease severity

Clinical picture	Supportive care	Antiviral treatment
**Asymptomatic infection**	None	None
**Mild case:** fever and/or asthenia with upper respiratory signs, not requiring supplemental oxygen	• Paracetamol (10–15 mg/kg every 4–6 h) in case of fever > 38 °C • Airway suction in case of obstruction	• Consider monoclonal antibodies only in the presence of risk factors^a^
**Moderate case:** respiratory signs/symptoms (such as cough, mild distress with polypnea) requiring supplemental oxygen with nasal cannulas or Venturi system +/−fever, difficulty in feeding, signs of dehydration	• Paracetamol in case of fever > 38 °C • Airway suction in case of obstruction • Oxygen therapy using nasal cannulas or facial mask with Venturi system (target oxygen saturation > 95%), refer to WHO Interim guidance • Intravenous access, adequate fluid and caloric intake based on hydration status • Monitor vital signs (Bedside-PEWS) [3] every 8 h (or before in case of changes in the clinical picture)	• Dexamethasone (0.1–0.2 mg/kg) or methylprednisolone (1–2 mg/kg day) • Remdesivir (5 mg/kg/1st day than 2.5 mg/kg for 5 days) • Dexamethasone/methylprednisolone plus Remdesivir
**Severe illness:** respiratory signs/symptoms (tachypnea, labored breathing) requiring supplemental oxygen with high flow nasal cannulas or non-invasive ventilation +/− fever, systemic signs of worsening (lethargy, inability to feed/drink)	• Paracetamol in case of fever > 38 °C • Airway suction in case of obstruction • Oxygen therapy using high-flow nasal cannulas or non-invasive ventilation (target oxygen saturation > 95%), refer to WHO Interim guidance • Intravenous access, adequate fluid and caloric intake based on hydration status. Monitor urinary output. • Venous thromboembolism prevention: low molecular-weight heparin • Avoid empiric antibiotic treatment if no evidence of bacterial infection (consult an infectious disease specialist or refer to hospital guidelines) • Monitor vital signs (Bedside-PEWS) [3] every 8 h (or before in case of changes in the clinical picture)	• Dexamethasone/methylprednisolone • Dexamethasone/methylprednisolone plus Remdesivir (available for this group of patients only within clinical trials)
**Critical illness:** ARDS Respiratory involvement requiring mechanical ventilation or extracorporeal membrane oxygenation	• Paracetamol in case of fever > 38 °C • Airway suction in case of obstruction • Oxygen therapy using mechanical ventilation (target oxygen saturation > 95%) or extracorporeal membrane oxygenation, refer to WHO Interim guidance • Intravenous access, adequate fluid and caloric intake based on hydration status. Monitor urinary output. • Venous thromboembolism prevention: low molecular-weight heparin • Avoid empiric antibiotic treatment if no evidence of bacterial infection (consult an infectious disease specialist or refer to hospital guidelines) • Monitor vital signs (Bedside-PEWS) [4] every 8 h (or before in case of changes in the clinical picture)	• Dexamethasone/methylprednisolone

^a^eligibility criteria for emergency use of mAb in high-risk adolescents between 12 and 17 years of age are the presence of

BMI >95th percentile for age and sex

Sickle cell disease

Congenital or acquired heart disease

Neurodevelopmental disorders (cerebral palsy)

Technological dependence (tracheostomy, gastrostomy, positive pressure ventilation (not related to Covid-19))

Asthma, reactive airways or chronic respiratory disease requiring daily medical supervision

In the last months the data from large multicenter trials conducted in adults have been published [6–14]. According to what is reported, current evidences suggest to avoid the use of hydroxychloroquine and lopinavir/ritonavir in patients with COVID-19, independently to the severity of the disease [6–8].

Trials conducted in adults showed a moderate efficacy of remdesivir in patients with supplementary low-flow oxygen requirement, but at present, results of randomized trials in children are not available [6, 10–13]. A consensus statement was issued by a panel of experts from the Pediatric Infectious Diseases Society, supporting the use of remdesivir in severe and critically ill children [14].

Nowadays, remdesivir is approved in children/adolescents older than 12 years of age with pneumonia requiring supplementary low flow-oxygen and, for these patients, it is delivered through the *Agenzia Italiana del Farmaco* (AIFA). In younger children remdesivir could be used within clinical trials or requested for a compassionate use: it should be started within 10 days since symptoms onset. A 5-days course of treatment is generally recommended, but it could be extended up to 10 days on a case-by-case basis. However, the available trials have not shown a significant clinical improvement with longer treatment [11, 12]. The suggested dose is 5 mg/kg intravenous (max 200 mg) the first day, followed by 2.5 mg/kg (max 100 mg) daily. At present, the dosage has not been established for the first 2 weeks of life and weight < 2.5 kg.

Results from the RECOVERY trial on dexamethasone showed a benefit of 6 mg daily dose in hospitalized adults with COVID-19 [9]. Equivalent dose of steroids (such as methylprednisolone 32 mg or prednisone 40 mg) is also suggested. However, data on steroid use in children are very limited. Therefore, steroids may be beneficial in pediatric patients with COVID-19 respiratory disease who require mechanical ventilation. The use of dexamethasone in patients who require other forms of supplemental oxygen support should be considered on a case-by-case basis [15]. On the contrary, there is no evidence supporting the use of oral or systemic steroids in outpatient children not requiring oxygen or respiratory support: it has no effects in reducing disease progression or hospital admission.

Novel virus-neutralizing monoclonal antibody therapies (bamlanivimab, bamlanivimab plus etesevimab and REGN-COV2) have been approved by the Food and Drug Administration. Moreover, other monoclonal antibodies are under investigation [16]. AIFA approved the emergency use of those drugs for patients older than 12 years of age, not hospitalized and not requiring supplementary oxygen, with recent onset of mild-moderate COVID-19 and at least one risk factor [17]. In particular, for the age group 12–17 years, the risk factors are the following: body mass index > 95 percentile for age, sickle cell anemia, congenital or acquired cardiac disease, neurodevelopment disease, device carriers (tracheostomy, gastrostomy, etc.), asthma or other respiratory disorders requiring daily treatment [17, 18]. Because of the limited experience, currently available only for adults and the reduced rate of hospitalization, the use of monoclonal antibodies in children/adolescents (older > 12 years) should be carefully evaluated case by case, considering the severity of the underlying condition. Moreover, some mutations of new variants of the SARS-CoV-2 virus may result in changes of the spike protein, that could interfere with the effectiveness of monoclonal antibodies [19].

The use of convalescent plasma in patients hospitalized with COVID-19 is still under debate. A randomized controlled trial on 228 patients did not show significant difference in overall mortality between patients treated with convalescent plasma or placebo [20]. Even the Italian Tsunami study on 487 patients enrolled in 27 clinical centres did not show a plasma benefit in terms of reducing the risk of respiratory worsening or death in the first 30 days [3].

In conclusion, the best treatment for pediatric COVID-19 is currently not well defined, because randomized trials in children are lacking. As new evidences are emerging, an updated treatment consensus may provide guidance to the clinicians for the management of children with SARS-CoV-2 infection.

## Data Availability

All the publication are listed in bibliography.
